# Exome sequencing identifies a novel *TTC37* mutation in the first reported case of Trichohepatoenteric syndrome (THE-S) in South Africa

**DOI:** 10.1186/s12881-017-0388-5

**Published:** 2017-03-14

**Authors:** Craig Kinnear, Brigitte Glanzmann, Eric Banda, Nikola Schlechter, Glenda Durrheim, Annika Neethling, Etienne Nel, Mardelle Schoeman, Glynis Johnson, Paul D. van Helden, Eileen G. Hoal, Monika Esser, Michael Urban, Marlo Möller

**Affiliations:** 1 SA MRC Centre for Tuberculosis Research, DST/NRF Centre of Excellence for Biomedical Tuberculosis Research, Division of Molecular Biology and Human Genetics, Faculty of Medicine and Health Sciences, Stellenbosch University, P.O. Box 241, Cape Town, 8000 South Africa; 2 National Health Laboratory Service, Immunology Unit, Division of Medical Microbiology, Department of Pathology, Tygerberg Hospital, Stellenbosch University, Cape Town, South Africa; 3 Department of Paediatrics and Child Health, Faculty of Medicine and Health Sciences, Stellenbosch University, Cape Town, South Africa

**Keywords:** Trichohepatoenteric syndrome, Primary immunodeficiency diseases, Exome, Consanguineous, Next generation sequencing, Sanger sequencing

## Abstract

**Background:**

Trichohepatoenteric syndrome (THE-S) or phenotypic diarrhoea of infancy is a rare autosomal recessive disorder characterised by severe infantile diarrhoea, facial dysmorphism, immunodeficiency and woolly hair. It was first described in 1982 in two infants with intractable diarrhoea, liver cirrhosis and abnormal hair structure on microscopy. We report on two siblings from a consanguineous family of Somali descent who, despite extensive clinical investigation, remained undiagnosed until their demise. The index patient died of fulminant cytomegalovirus pneumonitis at 3 months of age.

**Methods:**

Whole exome sequencing (WES) was performed on a premortem DNA sample from the index case. Variants in a homozygous recessive state or compound heterozygous state were prioritized as potential candidate variants using TAPER™. Sanger sequencing was done to genotype the parents, unaffected sibling and a deceased sibling for the variant of interest.

**Results:**

Exome sequencing identified a novel homozygous mutation (c.4507C > T, rs200067423) in *TTC37* which was confirmed by Sanger sequencing in the index case. The identification of this mutation led to the diagnosis of THE-S in the proband and the same homozygous variant was confirmed in a male sibling who died 4 years earlier with severe chronic diarrhoea of infancy. The unaffected parents and sister were heterozygous for the identified variant.

**Conclusions:**

WES permitted definitive genetic diagnosis despite an atypical presentation in the index case and suggests that severe infection, likely secondary to immunodeficiency, may be a presenting feature. In addition definitive molecular diagnosis allows for genetic counseling and future prenatal diagnosis, and demonstrates the value of WES for post-mortem diagnosis of disorders with a non-specific clinical presentation in which a Mendelian cause is suspected.

**Electronic supplementary material:**

The online version of this article (doi:10.1186/s12881-017-0388-5) contains supplementary material, which is available to authorized users.

## Background

Trichohepatoenteric syndrome (THE-S) is a rare autosomal recessive disorder characterised by severe infantile diarrhoea, intrauterine growth retardation (IUGR), hair abnormalities (mainly trichorrhexis nodosa), skin abnormalities (including café-au-lait macules), hepatopathy and immunodeficiency [1–5]. All affected children, who present with explosive diarrhoea, require parenteral nutrition for survival. In some instances parenteral nutrition is a life-long requirement, while in others the patient can be weaned to full enteral feeding [6]. Fine motor movement abnormalities and mental retardation have also been reported in some affected individuals [7]. The estimated prevalence of THE-S is approximately 1/400,000–500,000 live births [6] and since its first description in 1982 [1], less than 60 cases have been described, with cases being reported from Europe, Asia, the Middle East and North Africa.

The diagnosis should be suspected in the presence of the clinical triad of IUGR, severe protracted diarrhoea of early onset, and abnormal facial features with prominent ears and cheeks [7]. Additional features are often present but are less consistent. Even though these key syndromic features have been very well described, the age-related appearance of many of these features coupled with the estimated low prevalence of this disease make it challenging to recognize THE-S during early infancy [3, 7]. It should be noted that before the hair abnormalities, liver cirrhosis or facial dysmorphism become noticeable, many patients fail to thrive, and suffer from intractable diarrhoea and recurrent infections, which are three of the 10 warning signs of primary immunodeficiency diseases (PIDs) [8–11]. For these reasons, PIDs are often initially suspected in THE-S patients. In a review of the literature, Fabre and colleagues noted that 39 of 44 reported cases presented with an immunological defect [12]. These immunological deficits ranged from low serum immunoglobulin levels [12], deficits in antibody production following vaccinations [6], monoclonal hyper IgA [13] and low lymphocyte counts [1, 3].

While our understanding of the pathogenesis of THE-S remains limited, causal mutations in two genes, *TTC37* [6] and *SKIV2L* [7, 12, 14–16], both encoding components of the human Ski complex, have been reported. The Ski complex is an obligatory co-factor in the RNA exosome which is crucial for the accurate processing of nuclear RNA precursors and in the degradation of RNA in both the nucleus and the cytoplasm [17–19] . Most reported mutations are private and are distributed in all exons of *TTC37* and *SKIV2L*, but a founder mutation in *TTC37* (*c.2808G > A*) has been reported in patients from India [5].

Interestingly, one case of THE-S presenting with immunodeficiency without diarrhoea has recently been reported by Rider and co-workers [15]. Their study described a novel *TTC37* mutation in a patient who presented with frequent otitis media, viral infections, purulent conjunctivitis, woolly hair and distinctive facial features without the characteristic severe diarrhoea of infancy seen in THE-S [15].

Here we describe a patient with an atypical respiratory presentation in which exome sequencing identified a novel homozygous *TTC37* mutation and allowed the diagnosis of THE-S to be made.

## Methods

### Case description

**Fig. 1:**
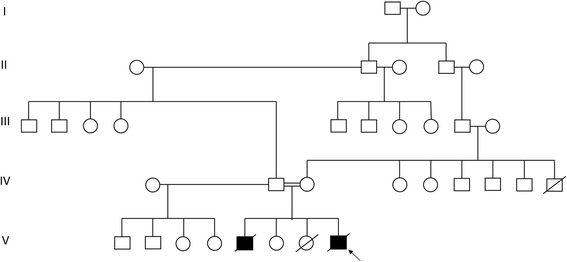
Multigeneration pedigree of the Trichohepatoenteric syndrome family

The index patient was a male of Somalian descent, born to consanguineous parents (first cousins once-removed, i.e. fourth degree relatives) living in South Africa (see Fig. 1 for pedigree). He was born at 35 weeks gestation, weighing 1940 g (3rd percentile) and remained in hospital initially for three weeks for neonatal jaundice and poor weight gain. The parents noted subtle patchy hyperpigmentation after he completed phototherapy for jaundice. His hair was shaved per their custom and was reportedly not unusual. On discharge he had received all routine vaccinations including live BCG and oral Polio drops.

**Fig. 2:**
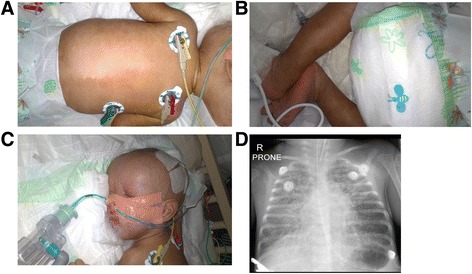
Clinical features of the proband. **a** Hyperpigmentation of the trunk. **b** Exfoliating rash present at the feet of the proband. **c** Prominent forehead with triangular appearance of the face. **d** Chest X-ray showing extensive bilateral bronchopneumonia

At age three months the patient presented with an acute history of respiratory distress. In addition, he had failed to thrive and his mass was 2600 g (<<3rd centile for corrected age). There was clinical evidence of a pneumonia and the liver edge was palpable 2 cm below the costal margin. Patchy, poorly circumscribed areas of hyperpigmentation were present on the trunk and limbs (Fig. 2a). There was an exfoliating rash particularly on the hands and feet with erythema of the palms and soles suggestive of erythroderma (Fig. 2b). The forehead was prominent (Fig. 2c), giving a triangular appearance to the face, but no hypertelorism or other dysmorphic features were noted.

An echocardiogram was performed and found to be normal, while chest x-ray showed an extensive bilateral interstitial pneumonitis with alveolar changes (Fig. 2d). The C-reactive protein (CRP) increased over 3 days from 9 to 43 mg/L (ref: 0–10). Tracheal aspirate was positive for cytomegalovirus (CMV) but not other respiratory viruses, and CMV viraemia was documented with a viral load of log 5.97. Blood, sputum and stool culture showed no evidence of bacterial infection. The full blood count was normal, with normal number of lymphocytes. On subset analysis, the natural killer (NK) cell count was 81/microliter (ref: 300–700) and the NK cell percentage was 3% (ref: 8–17) (Table 1). There was also a low serum IgG levels of <1.41 g/L (ref 3.00-10.00) but normal IgA and IgM. Liver functions showed very elevated gamma-glutamyl transferase (942 IU/L, ref: 2–30); somewhat elevated conjugated bilirubin (10 micromol/L, ref: 0–6), alanine transaminase (39 IU/L, ref: 4–35) and aspartate transaminase (75 IU/L, ref: 0–65); low albumin (23 g/L, ref: 28–46), and normal alkaline phosphatase (180 IU/L, ref: 75–316). The plasma amino acid profile was normal, as were urinary organic acids. Skin biopsy histology of a hyperpigmented macule showed abundant melanin but no specific features for pathological processes such as graft versus host disease.

**Table 1:** Phenotypic comparison of both siblings to literature

Clinical feature (HPO id)	Index case	Affected brother	Findings in 25 previously reported cases [1, 3, 4, 6, 15]
Consanguinity	Y	Y	13/22 (59%)
IUGR (HP:0001511)	Y	Y	23/25 (92%)
Preterm <37 wk (HP:0001622)	Y	N	12/25 (48%)
Dysmorphism			
Prominent forehead (HP:0011220)	Y	Y	20/20 (100%)
Hypertelorism (HP:0000316)	N	N	20/20 (100%)
Hypo/pigmented skin rash +/− erythroderma	Y	Y	6/20 (30%)
Unusual hair (Includes trichorrhexis nodosa, HP:0009886)	Unknown (shaved)	Y (‘sticking out’)	20/20 (100%)
Congenital heart defect (Abnormal heart morphology, HP:0001627)	N	N	5/20 (25%)
Musculoskelelal abnormality (Camptodactyly HP:0005617)	N	Y (camptodactyly)	1/12 (8%; Perthes disease)
Urinary tract abnormality (Hypospadias, HP:0000047)	N	Y (hypospadias)	3/20 (15%)
Gastrointestinal			
Chronic diarrhea (HP:0002028)	N	Y	24/25 (96%)
Failure to thrive (HP:0001508)	Y	Y (needed TPN)	25/25 (100%)
Villous atrophy on biopsy of small intestine (HP:0011473)	-	Y (patchy)	21/21 (100%)
Liver dysfunction or fibrosis	N	N	9/17 (53%, all with liver fibrosis)
Outcome			
Neurodevelopmental delay (HP:0012758)	?	Y	10/16 (63%)
Death	Y	Y	12/25 (48%)
If deceased, age at death	3 months	5 months	2-96 months
Immunological investigations {reference range}			
Lymphocyte count	2.53 {2.0–17.0 × 10^9^/l}	4.17 {2.0–17.0 × 10^9^/l}	Where reported was normal
% B-cells (CD19)	24% {19–31%}	20% {19–31%}	
% T-cells (CD3)	73% {58–67%}	72% {58–67%}	Weak T-cell proliferative response in some cases to antigens, not mitogens
% T-helper cells (CD4)	55% {38–50%}	39% {38–50%}	
% T-supressor cells (CD8)	16% {18–25%}	33% {18–25%}	
% Natural killer cells (CD16, 56) (Abnormality of NK cells HP:0012176)	3% {8–17%}	8% {8–17%}	
IgG level (IgG deficiency HP: 0004315)	<1.41 g/l {3.0–10.0 g/l}	1.96 g/l {3.0–10.0 g/l}	11/20 (55%) had ‘low immunoglobulins’. Other often had deficient vaccine response. Hence THE-S now in antibody deficiency group of the 2015 IUIS Primary Immunodeficiency classification
IgA level (IgA deficiency HP:0002720)	0.44 g/l {0.1–0.7 g/l}	<0.25 g/l {0.1–0.7 g/l}	
IgM level	0.92 g/l {0.2–1.10 g/l}	1.19 g/l {0.2–1.10 g/l}	

*HPO id* human phenotype ontology identification number, *Y* Yes, *N* No, *TPN* total parenteral nutrition, *NK* natural killer, *IUGR* intrauterine growth restriction, *IUIS* International Union of Immunological Societies

The patient was treated in the intensive care unit. Treatment included ventilation with a high frequency oscillator, intravenous ganciclovir, and other medications as appropriate. During the admission diarrhoea was noted, but was not a prominent feature. Following three weeks of intensive therapy, the patient died of ongoing severe pneumonitis. Supervening nosocomial sepsis was suspected but unproven. The family declined an autopsy. The clinical suspicion was of a combined immune deficit, with a family history that suggested an autosomal recessive or perhaps X-linked disorder.

A male sibling born 4 years earlier, weighed 1840 g (<<3rd centile) at term gestation. He was treated for neonatal jaundice and subsequently readmitted at two months of age with chronic diarrhoea, dehydration and failure to thrive. He was noticed to have hair that was “sticking out”, and both hyper- and hypopigmented skin macules. Other clinical features included a prominent forehead with a triangular face and low-set ears, penile hypospadias and bilateral camptodactyly of the fingers. Further laboratory investigations confirmed a secretory diarrhoea with albumin loss and metabolic acidosis. Endoscopic biopsies of the stomach and duodenum were performed. The stomach histology was normal, whereas the duodenal biopsy showed flattening and thickening of some villi, and preserved crypt architecture; in addition the lamina propria contained a sprinkling of lymphocytes and plasma cells. The liver function test was only minimally deranged. The full blood count, lymphocyte subsets and serum immunoglobulin profile were similar to that of the index case, except that the IgA was also low. He remained in hospital until his demise at 5 months of age, following documented episodes of *Klebsiella* and Enterococcal septicaemia. Additional investigations showed a normal karyotype, and normal profile of urine organic acids and urine and blood amino acids. No definitive diagnosis was made, and some details only became available to us after the death of the index case. Phenotypic comparisons between the index case and his deceased brother are shown in Table 1. The couple also has a healthy daughter, and a female stillbirth for reasons which were considered to be unrelated (Fig. 1).

Prior to demise of the index case, the parents were counselled on a possible genetic immunodeficiency and consented for blood samples from all live members of the family to be submitted for exome sequencing.

The study was approved by the Health Research Ethics Committee of Stellenbosch University (study no. N13/05/075). Parents gave informed consent to participate in the study, which included the genetic evaluation of their children. Additional informed consent was obtained from the parents to include photographs in this article. The study adhered to the ethical guidelines as set out in the “Declaration of Helsinki, 2013” [20]. Venous blood for DNA extraction and WES was drawn from the index case (1 ml) and both of his parents (5 ml). Blood was also drawn from the unaffected sister for genetic analysis. DNA was purified from blood using the Nucleon BACC3 Kit (Amersham Biosciences, Buckinghamshire, UK). DNA from a formalin-fixed duodenal biopsy sample of the previously deceased male sibling was extracted using the QIAamp® DNA FFPE Tissue Kit (Qiagen, Hilden, Germany).

### Exome capture and sequencing

Library preparation for sequencing was carried out using the Ion AmpliSeq™ Exome RDY Kit and the Ion Xpress™ Barcode Adaptors 1–16 Kit (Life Technologies, Carlsbad, California, United States). The DNA template for sequencing was prepared on the Ion Chef system using the Ion PI™ Hi-Q™ Chef Kit and the Ion PI™ Chip Kit v3. Sequencing was carried out on the Ion Proton™ (Thermo Fisher, Carlsbad, California, United States) at the Centre for Proteomic and Genomic Research (CPGR), Observatory, Cape Town, South Africa.

### Read mapping, variant detection and functional annotation

Sequences were aligned to the human reference genome, hg19 using Torrent Mapping Alignment Program (TMAP, version 4.4.11-1) in the ion-analysis workflow on the Torrent Suite (version 4.4.3). Base quality score recalibration, indel realignment and variant calling were performed using the variantCaller (version 4.4.3.3) plugin on the Torrent Suite and variant annotation was performed. Variant prioritization was performed using TAPER^TM^, a custom-designed, in-house method and variants in a homozygous recessive state or compound heterozygous state were prioritized as potential candidate variants [21]. In brief, TAPER™ 1) submits VCF files to an online variant caller, 2) removes all synonymous and non-frameshift indel variants, 3) removes all variants with a frequency of greater than 1% if present in the 1000 Genomes Project or Exome Sequencing Project 6500, 4) removes all variants with negative Genomic Evolutionary Rate Profiling (GERP) +++ scores, 5) removes all variants with Functional Analysis through Hidden Markov Models (FATHMM) scores greater than 0.1, 6) removes X and Y chromosome variants (if the pedigree does not indicate sex-linked inheritance) and lastly 7) identifies associated disorders for prioritised genes. Additionally, to validate findings obtained using TAPER™, variant filtering and prioritization was also done using Ion Reporter™ Software (version 5.0).

### Analysis of identity-by-descent and runs of homozygosity

Using custom scripts, the VCF files for both the mother and father were merged to create a multi-sample VCF file. This file was subsequently filtered for single nucleotide substitutions that were flagged “PASS” by a custom variant caller filter. Identity by descent (IBD) was calculated uing PLINK (http://zzz.bwh.harvard.edu/plink/) [22]. IBD was estimated in order to determine the degree of relatedness across the two individuals (PI(HAT)). This is calculated by using the inferred probabilities of individuals sharing an allele. Individuals may therefore share no alleles, one allele or both alleles. Following IBD, the runs of homozygosity were calculated to identify which segments of specific chromosomes are identical to one another. This allowed further inference of IBD as these segments were identical as a result of inheritance as opposed to sequence similarity.

### Sanger sequencing

A 239 base pair fragment containing the candidate variant was polymerase chain reaction (PCR)-amplified from genomic DNA of the index case, his affected brother, his unaffected parents and his unaffected sister using the following primers: TTC37F- 5′ AATCATAATCAGACACTACATCTGC-3′ and TTC37R- 5′ GCTCATAGTCATCTTTGGCATATAA. Each amplicon was bi-directionally sequenced using the BigDye® Terminator v3.1 Cycle Sequencing Kit (Perkin-Elmer, Applied Biosystems Inc., Foster City, California, USA.), followed by electrophoresis on an ABI 3130XL Genetic Analyzer (Perkin-Elmer, Applied Biosystems Inc., Foster City, California, USA). All automated DNA sequencing reactions were performed at the Central Analytical Facility at Stellenbosch University, Stellenbosch, RSA.

## Results

IBD and segmental sharing were calculated between the two parents of the index case. A PI(HAT) score of 0.082765 (Table 2) was calculated for the parents of the proband. This is within the range of what would be expected for first degree cousins. Moreover, eight runs of homozygosity across six chromosomes were identified in the proband (Table 3).

**Table 2:** Identity by descent scores (IBD) between the mother and father of the index case

FID1	IID1	FID2	IID2	Z0	Z1	Z2	PI(HAT)
PID027	Mother	PID027	Father	0.05379	0.04621	0.07311	0.082765

*FID* Family ID, *IID* Individual ID, *Z0* probability that individuals at a specific marker will share no alleles, *Z1* probability that individuals at a specific marker will share 1 allele, *Z2* probability that individuals at a specific marker will share 2 alleles

**Table 3:** The identification of runs of homozygosity between the mother and father of the index case

CHR	SNP1	SNP2	POS1	POS2	KB	NSNP	DENSITY (KB/NSNP)	PHOM	PHET
1	rs6077208	rs773530539	21798208	23743698	1945.49	116	16.771	0.603	0.017
2	rs558321757	rs23253874	25050977	33764286	8713.30	236	36.920	0.674	0.038
2	rs773745425	rs83697568	178415694	180846743	2431.04	123	19.764	0.789	0.008
**5**	**rs107007917**	**rs70038334**	**90719129**	**94953379**	**4234.25**	**151**	**28.041**	**0.314**	**0.508**
6	rs7451498	rs9384046	148981394	153173999	4192.60	163	25.721	0.662	0.131
14	rs10134181	rs898927	92280037	98263147	5983.11	244	24.520	0.297	0.637
16	rs4785195	rs1004299	49932806	54343840	4411.03	129	34.194	0.676	0.180
16	rs990813	rs12596363	59070616	64124789	5054.17	117	43.198	0.088	0.124

Bold font denotes ROH in which *TTC37* is localized. All calculations were done using the VCF file obtained for the WES. Stringent quality control parameters were implemented before identity by decent (IBD) and ROH were calculated. Note that because of missing calls, PHOM + PHET < 1

*SNP1* start of the SNP segment, *SNP2* end of the SNP segment, *POS1* start of the physical position of the segment (base pair), *POS2* end of the physical position of the segment (base pair), *NSNP* number of SNPs in the segment, *KB* physical length of the segment, *PHOM* proportion of sites homozygous, *PHET* proportion of sites heterozygous

The summary of the exome sequencing data for the index case and both his parents are presented in Table 4. Following filtering for rare or unreported variants a total of seven rare, homozygous variants were identified (Table 5). Following further analysis and database mining for gene-disease association, we identified a rare, low frequency homozygous missense mutation *c. 4507C > T* (rs200067423) in exon 42 of *TTC37* (NM_014639) which results in the substitution of an arginine with a cysteine at amino acid 1503 (p.R1503C) as a possible disease-causing variant. This is the first time that this variant has been identified in homozygous form and the read depth of this variant was 43X. This variant was found in one of the eight identified runs of homozygosity (Table 3). Additionally, using the American College of Medical Genetics this variant is classified as likely pathogenic pathogenic (One moderate and four supporting; PM2, PP1, PP2, PP3 and PP4) [23]. No other known disease-causing mutations were identified in *TTC37* or *SKIV2L*, both of which were 100% covered by WES. Additionally, no disease-causing mutation was identified on the X chromosome (Additional file 1: Tables S1 and S2).

**Table 4:** Summary of exome sequencing data for the index case and his parents

	Proband	Mother	Father
Total captured regions size	64 Mb	64 Mb	64 Mb
% of captured regions with coverage >10	99.7	99.7	99.8
Average coverage of captured region (%)	98.8	98.6	99.0
Total number of SNPs	21,858	22,433	22,043
Total number of INDELs	510	503	515
N rare homozygous	23	11	16
N rare heterozygous	269	201	201
N X linked	21	15	18

*SNP* single nucleotide polymorphism

**Table 5:** Shortlist of seven candidate variants identified as plausible disease-causing variants

Gene	SNV	dbSNP	1000 genomes project frequency						ExAC genome browser frequency								ESP6500 frequency		
			ALL	AFR	AMR	EAS	EUR	SAS	ALL	AFR	AMR	EAS	FIN	NFE	OTH	SAS	ALL	AA	EA
*KCTD1*	R336C	None	0.00	0.00	0.00	0.00	0.00	0.00	5.15e-05	0.00	0.00	0.00	0.00	0.0001	0.00	0.00	0.00	0.00	0.00
*COL6A5*	P349L	rs142949552	0.002	0.00	0.00	0.00	0.001	0.0092	0.0131	0.0005	0.0049	0.00	0.00	0.0039	0.0158	0.0279	0.00	0.00	0.00
*SH3TC1*	P778L	rs145110444	0.002	0.0053	0.00	0.001	0.001	0.001	0.0009	0.0056	8.78e-05	0.00	0.0003	0.0005	0.0012	0.0012	0.0022	0.0007	0.0003
*EVC2*	L502R	rs145909403	0.0006	0.0023	0.00	0.00	0.00	0.00	0.0001	0.0012	0.00	0.00	0.00	0.00	0.00	0.00	0.0002	0.0002	0.00
*TTC37*	R1503C	rs200067423	0.0002	0.0008	0.00	0.00	0.00	0.00	4.16e-05	0.0004	8.74e-05	0.00	0.00	0.00	0.00	0.00	0.0001	0.0002	0.00
*COL6A6*	E368G	None	0.00	0.00	0.00	0.00	0.00	0.00	0.00	0.00	0.00	0.00	0.00	0.00	0.00	0.00	0.00	0.00	0.00
*MFN2*	H20Y	rs201715603	0.00	0.00	0.00	0.00	0.00	0.00	0.0001	0.00	8.64e-05	0.00	0.00	0.0001	0.0011	0,0001	0.0001	0.00	0.0001

*KCTD1* Potassium Channel Tetramerization Domain Containing 1, *COL6A5* Collagen Type VI Alpha 5, *SH3TC1* SH3 Domain And Tetratricopeptide Repeats 1, *EVC2* EvC Ciliary Complex Subunit 2, *TTC37* Tetratricopeptide Repeat Domain 37, *COL6A6* Collagen Type VI Alpha 6, *MFN2* Mitofusin 2, *SNV* single nucleotide variant, *AFR* African, *AMR* Ad Mixed American, *EAS* East Asian, *EUR* European, *SAS* South Asian, *FIN* Finnish, *NFE* Non-Finnish European, *OTH* Other, *AA* African American, *EA* European American

**Fig. 3:**
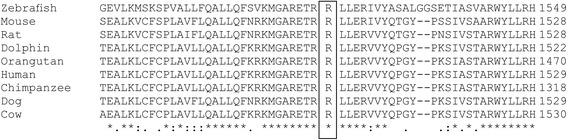
ClustalW multiple sequence alignment showing the highly conserved position of the *c.4507C > T* (p.1053R > C) mutation in *TTC37*

**Fig. 4:**
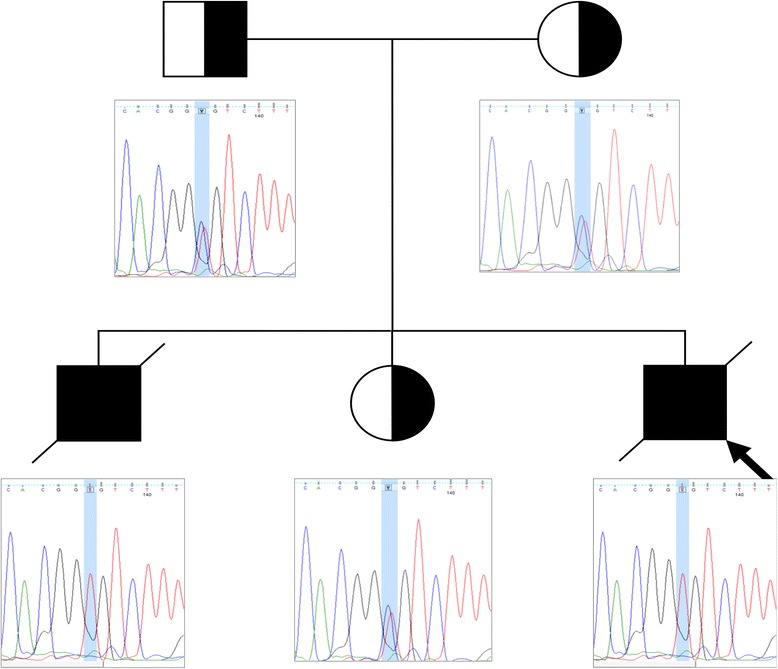
Validation of the mutation using Sanger sequencing. The proband (indicated by the black arrow) and affected deceased brother are homozygous for *TTC37 c.4507C > T* (p.1503R > C), while both unaffected parents as well as the unaffected sibling are heterozygotes

The presence of the variant in the patient and family members was verified using Sanger sequencing. The details of the mutation are summarised in Table 6. *In silico* predictions of the pathogenicity of the *TTC37 c. 4507C > T* mutation (SIFT, Polyphen) found that this mutation is damaging (Table 5). Multiple sequence alignment between species showed that the mutation occurs at a highly conserved residue (Fig. 3). Both parents as well as the healthy sister were heterozygous for the mutation, while the previously deceased sibling was homozygous (Fig. 4). This is the first reported case of THE-S in a patient of Somali descent as well as the first reported case in South Africa.

**Table 6:** Details of the candidate variant narrowed down using consecutive filters based on an autosomal recessive model of inheritance and low frequency

Chromosome	Chr 5
Position	94803683
Gene name	*TTC37*
RefSeq	NM_014639
Reference sequence	G
Proband: number of reads with reference	0
Proband: alternative	A
Proband: number of reads with alternative	43
Mother: number of reads with reference	88
Mother: alternative	A
Mother: number of reads with alternative	43
Father: number of reads with reference	49
Father: alternative	A
Father: number of reads with alternative	11
Mutation type	Missense
Mutation: DNA (HGVS nomenclature _c.)	c.4597 C > T
Mutation: protein (HGVS nomenclature _p.)	R1503C
Prediction < SIFT	Damaging
Prediction < PolyPhen-2	Probably damaging
Sanger verification	Yes

*HGVS* Human Genome Variation Society, *SIFT* Sorting Intolerant from Tolerant

## Discussion

THE-S is an extremely rare autosomal recessive disorder that typically manifests with severe, explosive diarrhoea in infancy, hair abnormalities, dysmorphic facial features and immunodeficiency. Here we describe brothers with a rare homozygous *TTC37* mutation (not previously described in the homozygous state), and presenting with many THE-S clinical features, but an atypical presentation in the index case. Due to this atypical presentation, and the lack of some of the specific features, THE-S was not initially considered. The characteristic hair abnormalities were not observed because his head was shaven. In addition, THE-S has not been previously diagnosed in South Africa, where consanguinity is uncommon.

The sibling of the index case had a more typical gastrointestinal presentation for THE-S, but the details of the case were not immediately available. In addition, the presence of hypospadias and camptodactyly would suggest consideration of other diagnostic possibilities. As a result, although a Mendelian disorder, either autosomal recessive or X-linked, was strongly suspected, the differential diagnosis remained broad despite quite extensive laboratory investigation.

Whole exome sequencing, conducted to clarify the clinical diagnosis, revealed a rare homozygous missense *c. 4507C > T* mutation (rs200067423) in *TTC37*, one of two genes previously implicated in THE-S in several independent investigations. The patient was found to be homozygous for the mutation while each of his consanguineous parents and his unaffected sister were heterozygous. We also identified the same homozygous mutation in his affected brother. This mutation results in a substitution of an arginine, which is a hydrophilic positively charged amino acid, with a hydrophobic uncharged cysteine residue at amino acid position 1503 of the protein. Based on the differences in charge and hydrophobicity between the arginine and cysteine and the fact that cysteine has the potential to form di-sulphide bonds, it is highly likely that the mutation leads to the loss of hydrogen bonds or impairs the proper folding of TTC37 [24]. Additionally, multiple species alignment shows that the mutation occurs at a highly conserved residue.

The findings on history, examination and laboratory investigations in both affected boys are consistent with a diagnosis of THE-S, despite the atypical presentation in the index case. IUGR is described as a consistent finding [7] and was present in both affected boys. The pre-deceased brother had chronic secretory diarrhoea with onset in early infancy, with associated failure to thrive, as described in the original article by Stankler et al.[1]. In the index case diarrhoea was present but less prominent than the severe respiratory infection.

On examination, a wide and prominent forehead is a consistent finding in THE-S [6] and was present in both cases. Skin changes occur frequently and include erythroderma [3] and patchy skin hyperpigmentation [7]. The index case had erythroderma and both had patchy hyperpigmentation. Trichorrhexis nodosa was not formally assessed, but the earlier sibling was known to have unusual-looking scalp hair. Both had neonatal jaundice and the index case had evidence of ongoing liver dysfunction, which is a common finding in THE-S [6]. The findings on duodenal histology for the earlier sibling were not very specific, but were consistent with previously reported findings in THE-S. Taken together, the results of the exome sequencing in the index case and the clinical findings in both affected siblings confirm a diagnosis of THE-S.

The fulminant CMV pneumonitis in the index case, taken together with the abnormal immunological profile, suggests underlying primary immunodeficiency. While humoral immune deficits are known to be common in THE-S [6, 14], the low natural killer (NK) cell count, more marked in the index case, has not been previously described. NK cells are important in recognizing and killing virally infected cells, and deficiency predisposes specifically to severe infections with herpes viruses such as CMV [25]. This provides a possible explanation for the severity of the CMV pneumonitis. Although childhood exposure to CMV is common, we found two prior cases of CMV hepatitis in previous reports of THE-S [3, 4], one of which occurred shortly after liver transplantation and therefore perhaps was secondary to use of immunosuppressants [4]. The predisposition to severe CMV may not be common in THE-S, but an alternative possibility is that THE-S is undetected in cases with early death.

## Conclusions

WES has been available for several years, but its usefulness as a routine clinical diagnostic tool is only now beginning to emerge [26, 27]. WES has particular value in diagnosing the cause of rare conditions with unclear phenotypes [28] or with an atypical presentation [29] as found in our index case. We demonstrate how WES, together with detailed phenotyping of a previously and more typically affected family member, led to a post-mortem molecular diagnosis of THE-S in an atypical index case. This allowed for genetic counselling and potential further genetic testing in the family; for carrier status, diagnosis or prenatal diagnosis. The findings in the index case broaden the range of phenotypes associated with THE-S to include presentation with fulminant CMV pneumonitis, and low NK cell count. We recommend that immunological investigations for known or suspected cases of THE-S include detailed immune function testing of antibody related function, but also careful screening for viral infections [10].

## Additional file

Additional file 1: Supplementary Tables. (DOCX 13 kb)

## Abbreviations

BCG: Bacillus Calmete-Guérin
CMV: Cytomegalovirus
CRP: C-reactive protein
DNA: Deoxyribonucleic acid
FATHMM: Functional Analysis through Hidden Markov Models
GERP: Genomic Evolutionary Rate Profiling
IBD: Identity by descent
IgA: Immunoglobulin A
IgG: Immunoglobulin G
IgM: Immunoglobulin M
IUGR: Intrauterine growth retardation
NK: Natural killer
PCR: Polymerase chain reaction
PID: Primary immunodeficiency diseases
SIFT: Sorting Intolerant From Tolerant
*SKIV2L*: Ski2 Like RNA Helicase
TAPER: Tool for Automated selection and Prioritization for Efficient Retrieval of sequence variants
THE-S: Trichohepatoentric syndrome
*TTC37*: Tetratricopeptide Repeat Domain 37
VCF: Variant call format
WES: Whole exome sequencing
